# An activin receptor-like kinase 1–governed monocytic lineage shapes an immunosuppressive landscape in breast cancer metastases

**DOI:** 10.1172/JCI183086

**Published:** 2025-01-14

**Authors:** Mehrnaz Safaee Talkhoncheh, Jonas Sjölund, Paulina Bolivar, Ewa Kurzejamska, Eugenia Cordero, Teia Vallès Pagès, Sara Larsson, Sophie Lehn, Gustav Frimannsson, Viktor Ingesson, Sebastian Braun, Jessica Pantaleo, Clara Oudenaarden, Martin Lauss, R. Scott Pearsall, Göran Jönsson, Charlotte Rolny, Matteo Bocci, Kristian Pietras

**Affiliations:** 1 Department of Laboratory Medicine, Division of Translational Cancer Research, Lund University Cancer Centre, Medicon Village, Lund University, Lund, Sweden.; 2 Department of Laboratory Medicine, Karolinska Institutet, Solna, Sweden.; 3 Lund University Diabetes Centre, Clinical Research Center, Lund University, Lund, Sweden.; 4 Biotech Research and Innovation Center, University of Copenhagen, Copenhagen, Denmark.; 5 Department of Clinical Sciences, Division of Oncology and Pathology, Lund University Cancer Centre, Lund University, Lund, Sweden.; 6 P2 Biopharma Consulting, North Reading, Massachusetts, USA.; 7 Department of Oncology-Pathology, Karolinska Institutet, Solna, Sweden.; 8 IO Biotech ApS, Copenhagen, Denmark.

**Keywords:** Immunology, Oncology, Breast cancer, Cancer immunotherapy, Endothelial cells

## Abstract

The biology centered around the TGF-β type I receptor activin receptor-like kinase (ALK) 1 (encoded by *ACVRL1*) has been almost exclusively based on its reported endothelial expression pattern since its first functional characterization more than 2 decades ago. Here, in efforts to better define the therapeutic context in which to use ALK1 inhibitors, we uncover a population of tumor-associated macrophages (TAMs) that, by virtue of their unanticipated *Acvrl1* expression, are effector targets for adjuvant antiangiogenic immunotherapy in mouse models of metastatic breast cancer. The combinatorial benefit depended on ALK1-mediated modulation of the differentiation potential of bone marrow–derived granulocyte-macrophage progenitors, the release of CD14^+^ monocytes into circulation, and their eventual extravasation. Notably, *ACVRL1*^+^ TAMs coincided with an immunosuppressive phenotype and were overrepresented in human cancers progressing on therapy. Accordingly, breast cancer patients with a prominent *ACVRL1*^hi^ TAM signature exhibited a significantly shorter survival. In conclusion, we shed light on an unexpected multimodal regulation of tumorigenic phenotypes by ALK1 and demonstrate its utility as a target for antiangiogenic immunotherapy.

## Abstract 2

<span>ALK1 targeting redirects the differentiation trajectory and recruitment of bone-marrow myeloid precursors, depriving the metastatic niche and the systemic macroenvironment of an immunosuppressive component. </span>

## Graphical abstract

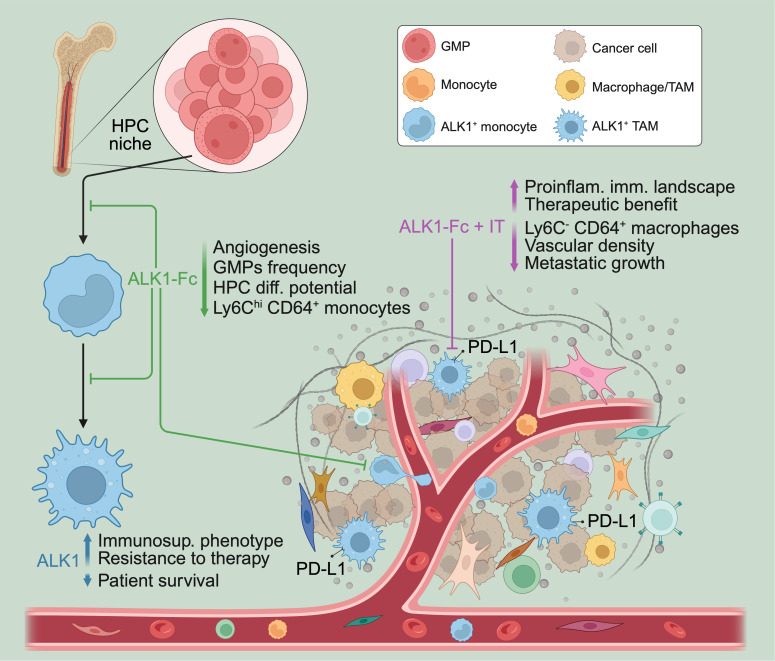

## Introduction

The activity of the TGF-β superfamily of ligands and receptors constitutes an essential component of physiological and developmental processes (1). In a similar fashion — owing to the pleiotropic effects of this complex pathway — the molecular cues instigated by TGF-β signaling are an established hallmark of cancer (2). Nonetheless, translation to clinical application of TGF-β pathway inhibitors has proved difficult, since the signal transduction cascade is cell type specific (3), environmentally controlled (4), and stage dictated throughout tumorigenesis (5). Knockout studies in mice have detailed the normal function of the TGF-β type I receptor activin receptor-like kinase (ALK) 1, encoded by *Acvrl1*, invariably resulting in embryonic lethality due to gross vascular alterations (6). Even though additional studies reported antithetic roles for ALK1 during physiological angiogenesis, a more generalized consensus has been attained in cancer, where ALK1 mediates proangiogenic stimuli linked to disease evolution (7, 8).

We and others have previously exploited targeting of ALK1 activity to impinge on tumor growth, either through genetic or pharmacological means (9–11), e.g., via the ligand trap RAP-041/dalantercept (referred to as ALK1-Fc from hereon). ALK1-Fc is a decoy receptor that traps the high-affinity ligands bone morphogenetic protein (BMP) 9 and 10, resulting in a moderated vascular tree and reduced tumor load (9, 12–14). In addition, upon ALK1 blockade, the pruned tumor-associated vasculature displays an increased pericyte coverage, conceivably contributing to a more efficient drug delivery and a resulting reduction of metastatic dissemination to the lungs (9, 13). Despite these promising results, the clinical endorsement of ALK1 inhibitors was short lived: indeed, following promising phase 1 trials in patients with heavily pretreated advanced solid cancers (15, 16), expansion and phase 2 tests with dalantercept, either alone or in combination with VEGF inhibitors, fell short of additional favorable effects, despite a tolerable safety profile (17–22). More recently, we determined that the network of genes correlated with *ACVRL1* expression is associated with a series of processes defining the tumor microenvironment (TME) across human solid malignancies, with a large overrepresentation of pathways linked to immune cell function and regulation (23). However, how ALK1 signaling in the tumor endothelium and beyond contributes to metastatic and drug-resistant properties of the local tumor milieu remains unexplored.

Here, by modeling adjuvant (i.e., postsurgical) therapy targeting ALK1 in experimental metastatic breast cancer, we characterize a previously overlooked subpopulation of tumor-associated macrophages (TAMs) identified by expression of *Acvrl1*. Through integration of in vivo platforms, ex vivo assays and longitudinal in silico data, we demonstrate that *Acvrl1*^+^ TAMs derived from circulating CD14^+^ monocytes that were recruited to the tumor site. Indeed, blunted ALK1 signaling directly shaped the potency of hematopoietic progenitor cells (HPCs) in the bone marrow by hindering their differentiation into the monocytic lineage as well as the subsequent mobilization of monocytes and their maturation to macrophages. Moreover, the emergence of *ACVRL1*^+^ TAMs was associated with disease progression upon development of therapeutic resistance in cancer patients. Consequently, high expression of a TAM-specific *ACVRL1*^hi^ signature identified patients with exceedingly poor survival in breast cancer. As a proof of concept, in vivo combination of ALK1 inhibition and immune checkpoint blockade resulted in substantially reduced metastatic disease. Finally, by means of 3D modeling of the immune-endothelial interface, FACS immunophenotyping, and multiplex imaging coupled with spatial analysis, we further uncovered the multimodal local and systemic effects of an impaired ALK1 signaling in the TME.

In conclusion, our work uncovers that ALK1 embodies a dual angiogenic and immunomodulatory function in breast cancer, thereby providing a rationale for the reevaluation of ALK1-blocking agents in combination with immune checkpoint blockade.

## Results

### Inhibition of ALK1 alters the extent of immune infiltrate in experimental primary and metastatic breast cancer.

Based on our previous observations that *ACVRL1* expression is associated with immune features of the TME in a series of human solid malignancies (23), we confirmed the generality of this regulation in breast cancer. As expected, the network of genes correlated with *ACVRL1* expression paralleled a similar pattern in the The Cancer Genome Atlas (TCGA) Breast Invasive Carcinoma cohort (24, 25), with a highly significant enrichment for angiogenesis and hypoxia, in keeping with the reported endothelial expression of ALK1 (Supplemental Table 1; supplemental material available online with this article; https://doi.org/10.1172/JCI183086DS1). However, the largest group of gene set enrichment analysis terms fell into a broad collection of processes defining the functionality and regulation of the immune cell compartment, e.g., IFN-γ response, IL2-STAT5 signaling, and TNF-α signaling via NF-κB (Supplemental Figure 1A and Supplemental Table 1).

**Figure 1:**
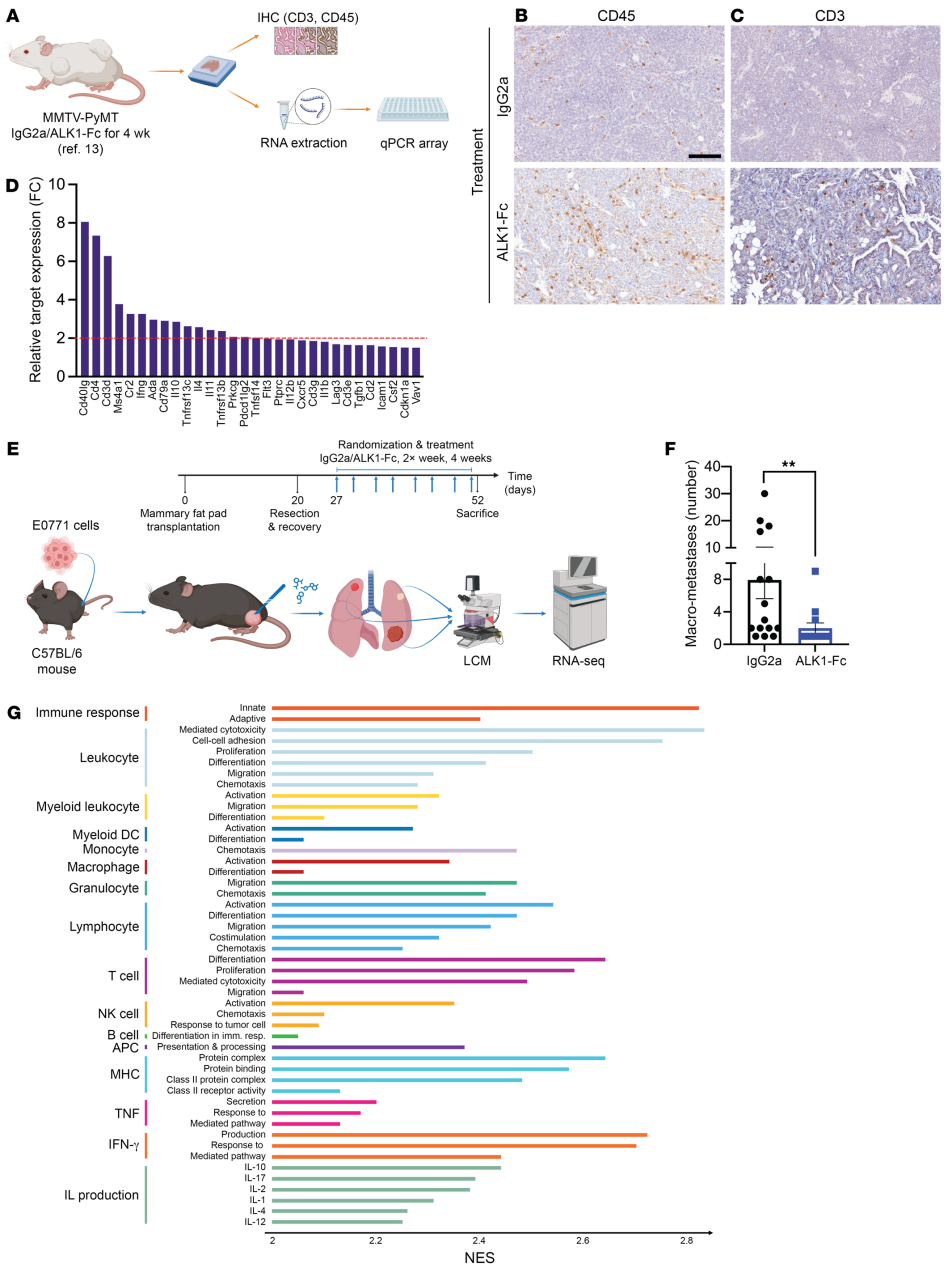
Inhibition of ALK1 alters the extent of immune infiltrate in experimental primary and metastatic breast cancer. (**A**) Analysis of archival breast cancer tissue from the transgenic MMTV-PyMT mouse model treated with IgG2a or ALK1-Fc (13). (**B** and **C**) Representative fields of IHC for CD45 (**B**), and CD3 (**C**), ALK1-Fc versus IgG2a (*n* = 4 for IgG2a, *n* = 6 for ALK1-Fc). Scale bar: 100 μm. (**D**) Plot displaying the fold-change expression of target genes from the qRT-PCR, ALK1-Fc versus IgG2a. F.C., fold change. (**E**) Experimental design of the adjuvant trial based on the orthotopic transplantation of 5 × 10^5^ E0771 cells in syngeneic C57BL/6 hosts (*n* = 15 for IgG2a, *n* = 13 for ALK1-Fc). (**F**) Quantification of macrometastases at sacrifice, ALK1-Fc versus IgG2a. Data are represented as mean with SEM. **P < 0.01, Mann-Whitney *U* test. (**G**) Selection of significant gene ontology terms (adjusted *P* < 0.05) from the analysis performed on bulk RNA-Seq of E0771 lung metastases. Normalized enrichment score (NES) values, ALK1-Fc versus IgG2a. APC, antigen presenting cell.

In light of these results, we immunostained experimental breast cancer tissue from transgenic MMTV-PyMT mice treated with ALK1-Fc (13) (Figure 1A). Assessment of IHC revealed an increased abundance of immunoreactivity against the broad leukocyte marker CD45 in ALK1-Fc–treated tumors compared with control IgG2a (Figure 1B). An analogous rise was observed for the T lymphocyte–restricted marker CD3 (Figure 1C). Furthermore, RNA was extracted from tissue sections from the same tumors and used as input for a quantitative reverse transcriptase PCR (qRT-PCR) array focusing on immune-related genes. As shown in Figure 1D and Supplemental Table 2, tumors exposed to ALK1-Fc displayed a substantial increase in the expression of genes broadly regulating the identity and activation state of different immune cell types, including *Cd40lg*, *Cd4*, *Cd3d*, *Ifng*, *Il4*, and *Pdcd1lg2* (PD-L2).

Primary tumors from MMTV-PyMT mice, or resulting from E0771 breast cancer cell transplantation, did not respond to either programmed cell death protein 1 (PD-1) or cytotoxic T-lymphocyte antigen 4 (CTLA-4) inhibitors alone, or in combination with ALK1-Fc (Supplemental Figure 1, B and C). To better reflect the clinical management of breast cancer patients, and acknowledging the potent immunosuppressive effect of primary tumors (26), we next modeled an adjuvant therapy setup (Figure 1E). Following the engraftment of E0771 breast cancer cells in the abdominal mammary fat pad of syngeneic recipient hosts, incipient tumors were surgically resected when they reached 13 mm in the largest diameter. Five to 7 days after surgery, mice were randomized to receive IgG2a or ALK1-Fc for up to 4 weeks, while being carefully monitored for signs of disease spread to the lungs. At the experimental endpoint, administration of ALK1-Fc significantly reduced the number of macrometastases per mouse, without affecting metastatic incidence (Figure 1F).

Based on the relative size and distribution of the metastases, lung lesions were either manually isolated or laser-capture microdissected, and further processed for bulk RNA-Seq. Enrichment analysis outlined an overrepresentation in tumors from ALK1-Fc–treated mice of pathways related to inflammatory response, complement, and allograft rejection, as well as apoptosis, whereas DNA repair was greatly underscored (Supplemental Figure 1D and Supplemental Table 3). Strikingly, the statistically significant ontology terms covered both the innate and the adaptive arms of the defense response, encompassing more specialized functions such as interleukin production, response to IFN-γ, activation, migration, and chemotaxis of a series of distinct immune cell types (Figure 1G and Supplemental Table 3). Jointly, these data suggest that ALK1 inhibition promotes an inflammatory TME that may be further exploited for improved tumor control.

### Murine and human macrophages express Acvrl1/ACVRL1.

**Figure 2:**
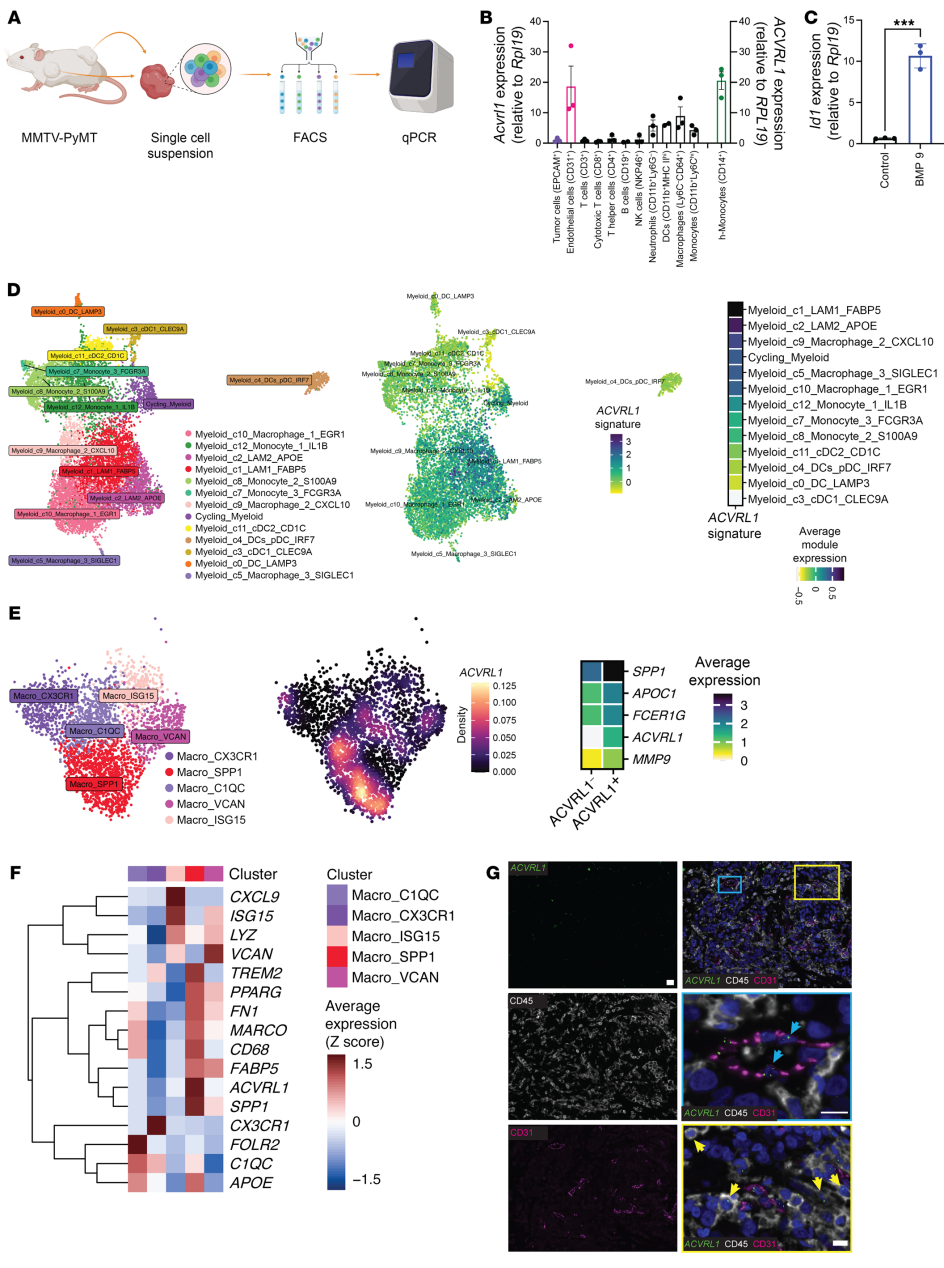
Murine and human macrophages express *Acvrl1*/*ACVRL1*. (**A** and **B**) Study design (**A**) for the quantification of *Acvrl1* expression in FACS-sorted immune cell populations from the MMTV-PyMT model (3 pooled experiments) (**B**). Positive (CD31^+^ endothelial cells) and negative (EpCAM^+^ epithelial cells) controls highlighted in magenta and purple, respectively. Expression of *ACVRL1* in freshly isolated human CD14^+^ monocytes from healthy donors (green). Data are represented as mean with SEM. (**C**) Expression of *Id1* in unstimulated (control) versus BMP9-stimulated BM-derived macrophages (representative of 3 independent experiments). Data are represented as mean with SEM. ***P < 0.001, unpaired, 2-tailed Student’s *t* test. (**D**) Overlay of a TAM-specific *ACVRL1* signature onto myeloid cells of a human breast cancer scRNA-Seq dataset (32). (**E**) Expression of *ACVRL1* in TAMs from the scRNA-Seq atlas of immune phenotypes (29, 30). Density plot of the expression of *ACVRL1* in TAMs. The average expression of the genes composing the TAM signature is presented in the heatmap for *ACVRL1*^+^ and *ACVRL1^–^* TAM populations. (**F**) Heatmap of the expression of *ACVRL1*, cluster markers, and prototypical TAM markers in the scRNA-Seq atlas of immune phenotypes (29). (**G**) Dual RNAscope ISH coupled with mIHC in human breast cancer. The protein markers CD31 (magenta) and CD45 (white) were used to describe the cellular distribution of the *ACVRL1* probe (green). Scale bars: 20 μm; 10 μm (inlet). Two inlets were annotated to highlight endothelial (cyan inlet/arrows) or immune-restricted accumulation of *ACVRL1* (yellow inlets/arrows).

The exclusive enrichment for immune processes following ALK1 inhibition made us wonder about the etiology of this regulation. Although an altered angiocrine signaling caused by inhibition of ALK1 is a logical and expected possibility considering the reported endothelial specificity of expression, it is tempting to speculate that a population of immune cells may directly react to ALK1 modulation, given the scale of the response observed at the gene expression level. To test the latter hypothesis, primary tumors from MMTV-PyMT mice were dissociated to single-cell suspensions, and expression of *Acvrl1* was queried by qRT-PCR of RNA isolated from immune cell populations sorted by flow cytometry (Figure 2A and Supplemental Figure 2A). Isolated endothelial cells and malignant cells served as positive and negative controls for *Acvrl1* expression, respectively (Figure 2B). Strikingly, as shown in Figure 2B, a subset of myeloid cells readily expressed *Acvrl1*, with Ly6C^–^CD64^+^ macrophages, CD11b^+^Ly6C^hi^ monocytes, CD11b^+^MHCII^+^ dendritic cells, and CD11b^+^Ly6G^–^ neutrophils displaying the highest expression levels. Additionally, the expression of *Acvrl1* bore functional implications, as stimulation of BM-derived macrophages with the high-affinity ligand BMP9 significantly upregulated the expression of the downstream target gene *Id1* (Figure 2C).

Scrutiny of the recently published genomic catalogue of the adult human breast (27) consolidated the expression of *ACVRL1* in a series of annotated myeloid cell types (Supplemental Figure 2B), including a variable yet conserved proportion (4%–8%) of macro-M2, macro-lipo, and macro-IFN subsets as the fractions with the highest expression, indicating that *ACVRL1* is a feature of several macrophage phenotypes and states. Moreover, our assessment of the expression of *ACVRL1* by means of qRT-PCR revealed prominent mRNA expression in freshly isolated human CD14^+^ monocytes from the peripheral blood of healthy donors (Figure 2B). In agreement with this finding, *ACVRL1* was also discernible in CD14^+^ monocytes in a single-cell RNA-Seq (scRNA-Seq) collection of the human BM (28) (Supplemental Figure 2C).

In the context of cancer, we started off by generating and validating a TAM-specific *ACVRL1* signature. Significantly differentially expressed genes (DEGs) between *ACVRL1*^+^ and *ACVRL1^–^* macrophages were extracted from a breast-specific map of immune phenotypes (29) with an updated cellular annotation (30) (Supplemental Table 4). This list was further filtered through a triple-negative breast cancer (TNBC) scRNA-Seq dataset (31) by applying a stringent criteria selection (i.e., high expression restricted to the macrophage cluster; Supplemental Table 5), leading to 4 genes: *SPP1*, *APOC1*, *FCER1G*, and *MMP9*. The final signature — which included these 4 genes as well as *ACVRL1* — teased out a distinct cluster of myeloid cells and TAMs when imposed on 2 additional breast cancer scRNA-Seq metadata (31, 32) (Supplemental Figure 2, D and E), with the highest average signature expression in lipid-associated macrophages (LAMs) (Figure 2D). Next, we queried the approximately 400 DEGs between *ACVRL1*^+^ versus *ACVRL1^–^* TAMs in relation to the hallmarks of intratumoral heterogeneity that were recently mapped out (33). Within the macrophage cell type, this analysis confirmed a significant enrichment for lipid-related, glycolysis, and proteasomal degradation metaprograms (Supplemental Figure 2F and Supplemental Table 6).

When closing in on TAMs, inspection of the breast-restricted immune atlas revealed the highest expression of *ACVRL1* in angiogenesis-associated, protumorigenic *SPP1*^+^ TAMs (34) (Figure 2E and Supplemental Figure 2G). Reassuringly, the average expression of the genes included in the signature was higher in *ACVRL1*^+^ versus *ACVRL1^–^* TAMs (Figure 2E). Within the *SPP1*^+^ cluster, *ACVRL1* expression strongly mirrored other immunosuppressive markers, such as *FABP5* and *TREM2*, with both genes further converging into the previously reported LAM phenotype (32, 35) (Figure 2F). To expand on the predicted recruited origin (and supported by *ACVRL1* expression in CD14^+^ monocytes), our analysis highlighted the distinct expression pattern of *ACVRL1* relative to *FOLR2*, which unequivocally identifies resident APOE^+^ TAMs (36); indeed, *FOLR2* expression exclusively segregated in the *C1QC*^+^ TAM cluster (Figure 2F).

Finally, to validate these results in patient material, the expression of ALK1 was probed in human breast cancer specimens by RNAscope (due to the paucity of antibodies specific for ALK1 suitable for immunostaining) coupled with highly sensitive multiplexed immunohistochemistry (mIHC) to identify constituent cell types. As expected, the signal of *ACVRL1* readily overlapped with the endothelial marker CD31 (cyan inlet/arrows, Figure 2G). Moreover, the characteristic dots of the RNA in situ hybridization (RNA-ISH) clearly accumulated in CD45-positive cells in the tissue (yellow inlet/arrows).

Taken together, our data reveal that expression of ALK1 is not, as previously reported, exclusive for the endothelium and that a population of recruited TAMs is also characterized by *ACVRL1* expression across human breast malignancies.

### ACVRL1-expressing TAMs display an immunosuppressive phenotype associated with resistance to therapy and poor survival.

**Figure 3:**
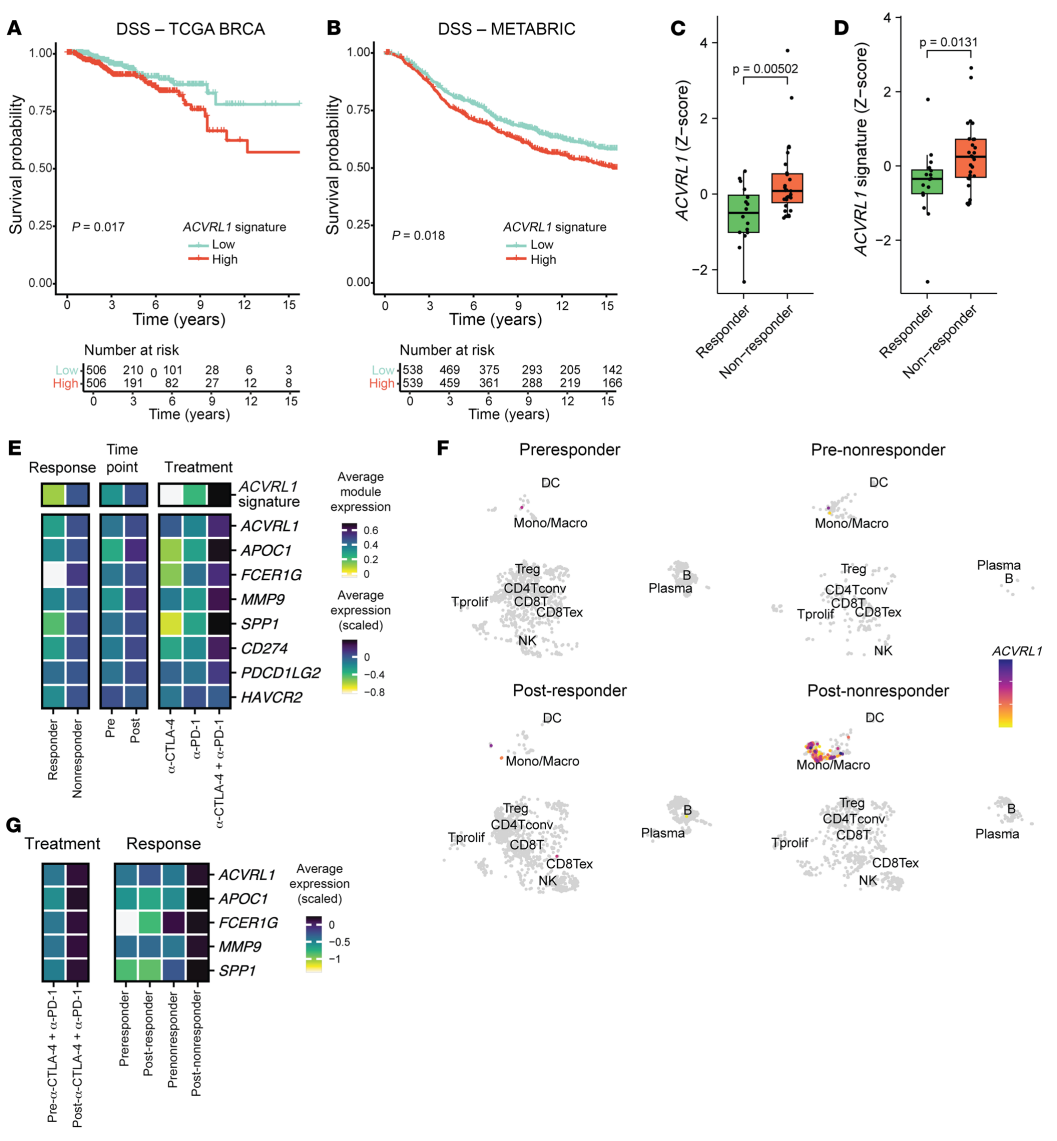
ACVRL1-expressing TAMs display an immunosuppressive phenotype associated with resistance to therapy and poor survival. (**A** and **B**) Survival analysis in the TCGA BRCA (37) (**A**) and METABRIC (38) (**B**) datasets. Patients were stratified into 2 risk groups based on the median value of the mean expression of a TAM-specific *ACVRL1* signature. The Kaplan-Meier curves show the DSS probabilities of the high (red) and low (green) signature expression groups in the 2 cohorts. *P* value: log-rank test. The tables summarize the relative Cox’s proportional hazard model analysis for each cohort. (**C** and **D**) Box plots depicting the expression of *ACVRL1* (**C**) and the 5-gene signature of *ACVRL1*^+^ macrophages (**D**) in a bulk RNA-Seq dataset of 43 TNBC patients sequenced before treatment with anti–PD-1 (41). Pretreatment features were then correlated to response to therapy (responders, *n* = 16; nonresponders, *n* = 27). Statistical analysis was performed using Wilcoxon’s rank sum test, and the *P* values were corrected for multiple testing with the Benjamini-Hochberg method. (**E** and **F**) Expression of *ACVRL1* in a CD45^+^-restricted scRNA-Seq compendium of 48 melanoma patients treated with immune checkpoint inhibitors (40). The average expression of the 5-gene signature, and the average scaled expression of the individual genes are presented in a heatmap (**E**) based on response, time point, and treatment arm. The average expression of *ACVRL1* in the combined CTLA-4 and PD-1 inhibition group was imposed on the UMAP, and further split to create 4 different groups: preresponder, postresponder, prenonresponder, and postnonresponder (**F**). Contingent on the aggregated data points in **F**, the average scaled expression of the 5 genes comprised in the *ACVRL1* signature is presented in a heatmap (**G**).

To gain additional insight into the translational relevance of the molecular cues instigated by ALK1 in macrophages, we estimated survival outcomes in 1,097 breast cancer patients from the TCGA repository (37) through Kaplan-Meier survival fractions, as well as a Cox’s proportional hazard model. After adjusting for stage, age at diagnosis, and estrogen receptor (ER) status, the *ACVRL1* signature*^hi^* group exhibited worse disease-specific survival (DSS) (*P* = 0.017, HR = 1.86 [95% CI = 1.14–3.03]; Figure 3A and Table 1), as well as progression-free interval (PFI) (*P* = 0.014, HR = 1.66 [95% CI = 116–2.38]; Supplemental Figure 3A), compared with the group of patients with the lowest *ACVRL1* signature expression; a similar trend was observed for overall survival (OS) (*P* = 0.19, HR = 1.23 [95% CI 0.87–1.75]; Supplemental Figure 3B). These results were validated in the METABRIC (38) dataset, which confirmed the significance for DSS (*P* = 0.018, HR = 1.34, 95% CI= 1.11–1.60; Figure 3B and Table 2), and the trend for OS (*P* = 0.077, HR = 1.14 [95% CI = 0.99–1.31]; Supplemental Figure 3C).

Prompted by these results, we set out to determine how myeloid expression of ALK1 tied in with the clinical performance of immunotherapy (IT). In a cohort or 43 TNBC patients, in which the pretreatment bulk tumor transcriptional pattern was correlated to the outcome of anti–PD-1 therapy (39), baseline expression of either *ACVRL1* alone, or of the gene signature for *ACVRL1*^+^ macrophages, was significantly higher in nonresponders (*n* = 27) versus responders (*n* = 16) (Figure 3, C and D), indicative of an immunosuppressive environment. However, limited by the evident lack of well-annotated datasets exploring predictive biomarkers for IT in breast cancer and the delay in including IT in standard of care for this malignancy (40), we resorted to melanoma, where the use of immune checkpoint inhibitors has revolutionized the clinical management of patients. Thus, we screened a cohort of 48 melanoma patients that were longitudinally sampled throughout therapy (41), in this case a regimen of anti–CTLA-4, anti–PD-1, or combined CTLA-4 and PD-1 blockade. The analysis of this dataset confirmed that ALK1-expressing macrophages represented a minor subset of TAMs at baseline (circa 7%), which were efficiently removed by therapy in the responder group (Supplemental Figure 3D). Strikingly, ALK1^+^ TAMs emerged prominently upon treatment resistance, where they represented approximately 18% of the tumor monocyte/macrophage cluster in the nonresponder group (Supplemental Figure 3E). Accordingly, the expression of the *ACVRL1* signature — as well as the individual genes included in it — was significantly higher in nonresponders versus responders and in post- versus pretreatment data points (Figure 3E and Supplemental Figure 3E). Notably, the highest frequency and average expression of *ACVRL1* were detected in the treatment arm combining dual CTLA-4 and PD-1 blockade. In light of this specific modulation, we homed in on the combined anti–CTLA-4 and anti–PD-1 group, and the data from this treatment arm were dichotomized based on response at each time point. As shown in Figure 3, F and G, the *ACVRL1*^+^ myeloid cells present at baseline persisted throughout therapy and further expanded in the nonresponders. This regulation was even more striking when considering the *ACVRL1* signature, which showed an inherent difference already at baseline (Supplemental Figure 3E), suggesting that components of this signature might be responsible for intrinsic, primary resistance to IT. Moreover, *ACVRL1* expression was tied to that of immunosuppressive markers such as *CD274* (PD-L1) and *PDCD1LG2* (PD-L2) in nonresponders in the global cohort (Figure 3C). These data imply that ALK1 signaling may be directly involved in the specification of an immunosuppressive phenotype in TAMs. In agreement with this hypothesis, a distinct peak for SMAD5, the signaling mediator downstream of ALK1, could be extrapolated in the promoter region of *CD274*/PD-L1 from ChIP-Seq data of hematopoietic tissue in the ENCODE database (42, 43), further intersecting with a domain of euchromatin, indicative of direct accessibility to transcription factors (Supplemental Figure 3F). This analysis revealed the specificity of the signal for SMAD5, as the DNA-binding and the histone mark patterns could not be replicated for *PDCD1LG2*/PD-L2 or *HAVCR2*/TIM-3 (Supplemental Figure 3, G and H). Collectively, these results offered us a rationale to combine ALK1 blockade with immune checkpoint inhibitors.

### Inhibition of ALK1 potentiates IT.

**Figure 4:**
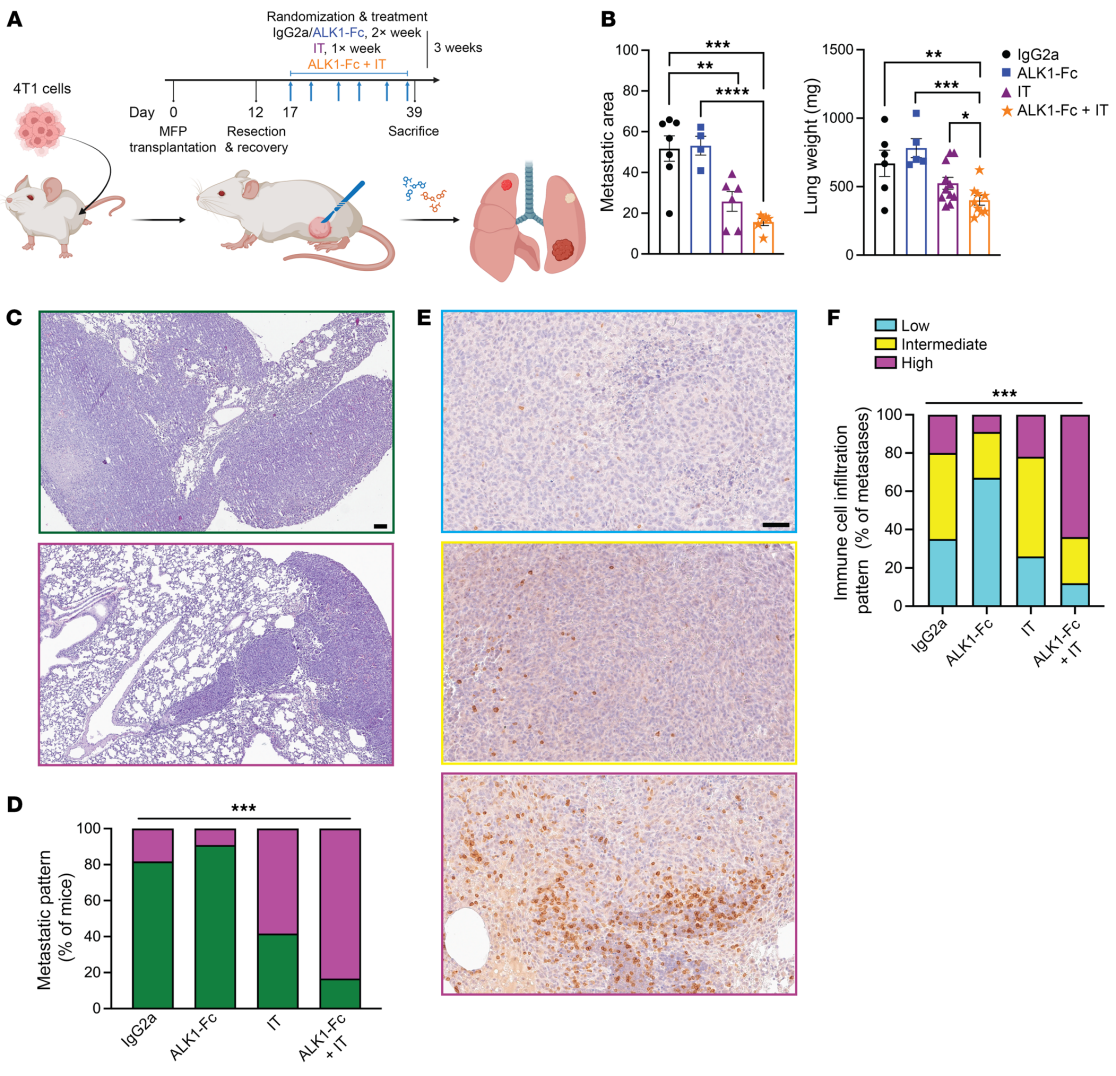
Inhibition of ALK1 potentiates IT. (**A** and **B**) Experimental design of the adjuvant trial based on the orthotopic transplantation of 5 × 10^4^ 4T1 cells in syngeneic BALB/c hosts (*n* = 7 for IgG2a, *n* = 4 for ALK1-Fc, *n* = 6 each for IT and ALK1-Fc + IT) (**A**). IT consists of a dual inhibition of PD-1 and CTLA-4. MFP, mammary fat pad. Quantification of metastatic area in the lungs in the different treated cohorts (**B**). Data are represented as mean with SEM. *P < 0.05; **P < 0.01; ***P < 0.001, 1-way ANOVA with Bonferroni’s post hoc test for the comparisons between ALK1-Fc versus ALK1-Fc + IT and IT versus ALK1-Fc + IT. (**C** and **D**) H&E staining of whole lung sections from the different cohorts in the 4T1 adjuvant trial. Representative pictograms of complete lung metastatic infiltration (green; **C**) or partial metastatic outgrowth (magenta), quantified in **D**. Scale bar: 100 μm. *P* value: χ^2^ test. (**E** and **F**) IHC for CD3 in whole lung sections from the different cohorts (**E**), and quantification of the proportions of the CD3 distribution in the metastases (**F**). The staining pattern was arbitrarily categorized as low (cyan), medium (yellow), and high (magenta). Scale bar: 50 μm. *P* value: χ^2^ test.

Motivated by the previous results indicating an ALK1-dependent specification of a protumorigenic immune state, we yet again exploited our resection-based adjuvant therapy pipeline, this time using the syngeneic 4T1 mammary carcinoma cell line. In an initial trial modeling a premetastatic setting, treatment with ALK1-Fc only showed a trend toward controlling disease progression, possibly reflecting the more aggressive nature of the 4T1 cell line (Supplemental Figure 4, A and B). Next, in a more advanced stage experimental setup, mice were randomized to receive postsurgery therapy with control IgG2a, ALK1-Fc, IT comprising dual PD-1 and CTLA-4 inhibition, or a combination of ALK1-Fc and IT (Figure 4A). At sacrifice, the total lung weight was recorded as a readout for metastatic burden. Notably, the cohort of mice that received combined treatment with ALK1-Fc and IT exhibited the lowest metastatic load of all groups, with a significantly reduced percentage of the lung composed of metastatic lesions, compared with ALK1-Fc alone (Figure 4B).

We corroborated these results by assessing the lung parenchyma in the different cohorts. More than 80% of the mice treated with either IgG2a or ALK1-Fc bore metastases that virtually invaded the whole lungs (Figure 4, C and D). Conversely, approximately 60% of the IT group and 85% of the animals in the ALK1-Fc + IT group exhibited distinct malignant lesions that were clearly encapsulated within adjacent/normal lung tissue (Figure 4, C and D). In line with this, evaluation of the distribution of CD3 staining within metastatic lesions supported that control tumors were more generally immune excluded (44, 45) and that they became increasingly more inflamed and readily infiltrated by lymphocytes as IT or a combination of ALK1-Fc + IT were administered (Figure 4, E and F). Even though processing of bulk RNA-Seq did not reveal major transcriptional differences between the cohorts, a series of specific genes with a clear involvement in angiogenesis and immune cell modulation, including *Dysf*, *Ly6g2*, *Ccl8*, and *Lrrc32*, were significantly up- or downregulated in the combination treatment group versus either monotherapy (Supplemental Figure 4, C–E, and Supplemental Table 7).

### Inhibition of ALK1 elicits local and systemic effects on the immune landscape.

**Figure 5:**
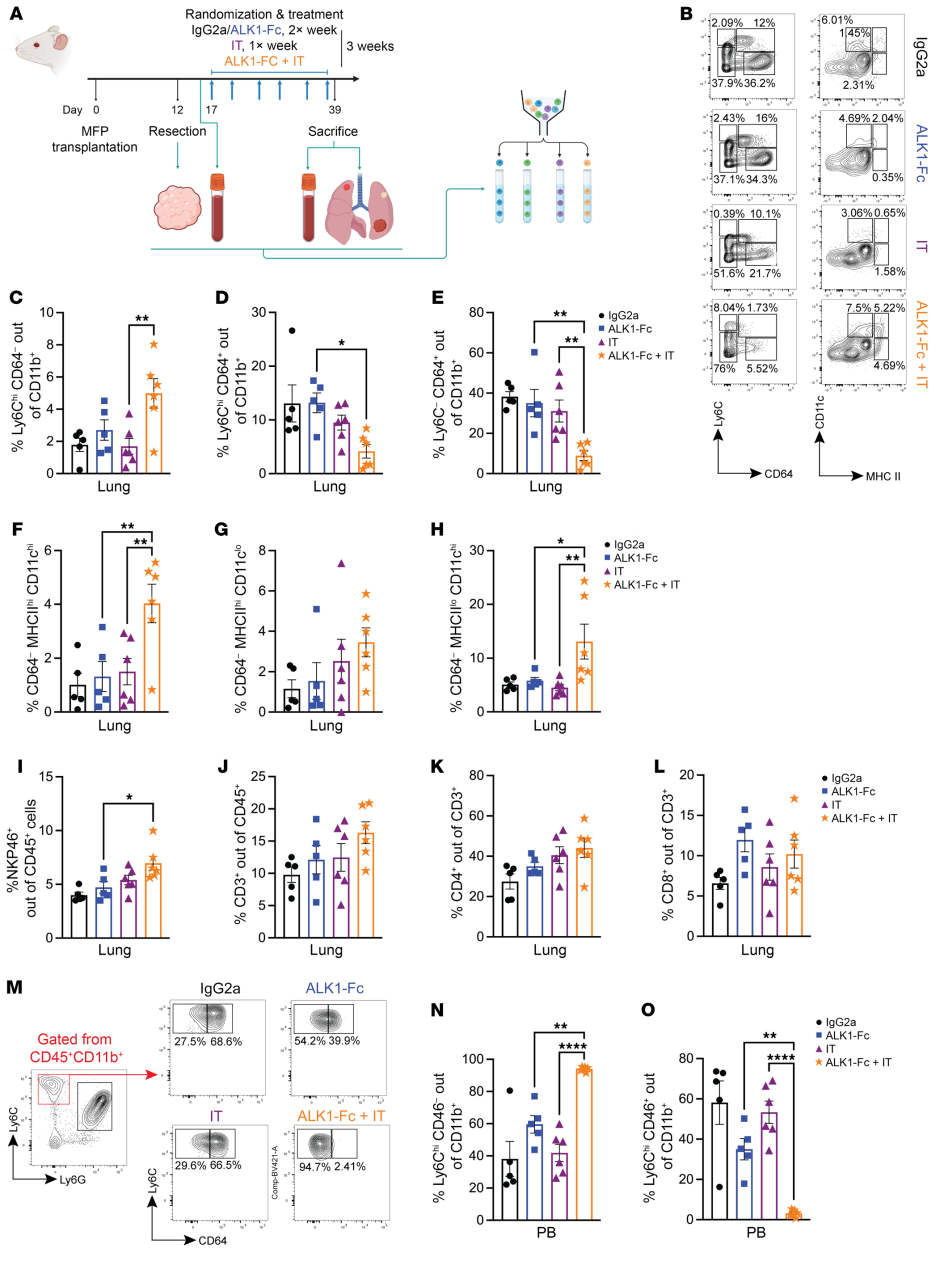
Antiangiogenic IT elicits tumor-specific and systemic effects on the immune cell composition. (**A**) Experimental design of the 4T1-based adjuvant trial to study different population of myeloid and lymphoid cells via FACS. (**B**) Representative FACS plots for the myeloid compartment in the different cohorts (*n* = 5 each for IgG2a and ALK1-Fc, *n* = 6 each for IT and ALK1-Fc + IT). (**C** and **D**) From the CD11b^+^ gating, relative abundance of monocytes in lung tissue: Ly6C^hi^CD64^–^ (**C**), and Ly6C^hi^CD64^+^ (**D**). Data are represented as mean with SEM. *P* value: unpaired, 2-tailed *t* test. (**E**) From the CD11b^+^ gating, relative abundance of Ly6C^–^ CD64^+^ macrophages in lung tissue. (**F**–**H**) From the CD64^–^ gating, relative frequency of dendritic cells in lung tissue: MHCII^hi^CD11C^hi^ (**F**), MHCII^hi^CD11C^lo^ (**G**), and MHCII^lo^CD11C^hi^ (**G**). (**I**–**L**) From the CD45^+^ population, relative frequency of NKP46^+^ NK cells (**I**), CD3^+^ T cells (**J**), CD4^+^ T helper cells (**K**), and CD8^+^ CTLs (**L**). (**M**–**O**) From the CD45^+^CD11b^+^ cells, purity check of circulating monocytes in peripheral blood (**M**). Relative frequency of circulating monocytes: Ly6C^hi^CD64^–^ (**N**) and Ly6C^hi^CD64^+^ (**O**). Data are represented as mean with SEM. *P < 0.05; **P < 0.01; ****P < 0.0001, 1-way ANOVA with Bonferroni’s post hoc test for the comparisons between ALK1-Fc versus ALK1-Fc + IT and IT versus ALK1-Fc + IT.

Next, we sought to define which immune cell type is responsible for the integration of the signals coming from the different inhibitory cues during antiangiogenic IT. For this purpose, resected 4T1 primary tumors, lung metastases, and peripheral blood from a second equivalent experiment were used as input for a multicolor FACS immunophenotyping approach devised to dissect the lymphoid and myeloid arms of the immune system (46) (Figure 5, A and B). Combined treatment with ALK1-Fc and immune checkpoint blockade directly acted on monocytes and macrophages in the metastatic tissue, with a significantly increased frequency of Ly6C^hi^CD64^–^ and a concomitant reduction of Ly6C^hi^CD64^+^ cells (Figure 5, C and D). In line with this, the combined treatment also significantly lowered the proportion of CD64^+^ macrophages in the lungs (Figure 5E), and increased several subsets of dendritic cells (Figure 5, F–H). In light of the observed expression pattern of ALK1, the combined treatment indirectly altered the lymphoid landscape (Supplemental Figure 5, A–D) by significantly increasing the frequency of NK cells (Figure 5I), CD3^+^ T cells (Figure 5J), CD4^+^ T helper cells (Figure 5K), and a trend for CD8^+^ cytotoxic T lymphocytes (CTLs) (Figure 5L). Moreover, these intrametastatic changes were coupled with systemic alterations in peripheral blood composition (Supplemental Figure 5, E–I), where ALK1-Fc + IT remarkably stalled the differentiation from CD64^–^ to CD64^+^ monocytes (Figure 5, M–O). This effect appeared to be reliant on ALK1 signaling, as the same modality of regulation was also detected in the ALK1-Fc cohort.

Altogether, these data suggest a dynamic regulation at multiple levels in the hematopoietic differentiation cascade and the metastatic immune landscape by the addition of ALK1-Fc to an IT regimen.

### Vascular immune features reflect differential response to antiangiogenic IT.

**Figure 6:**
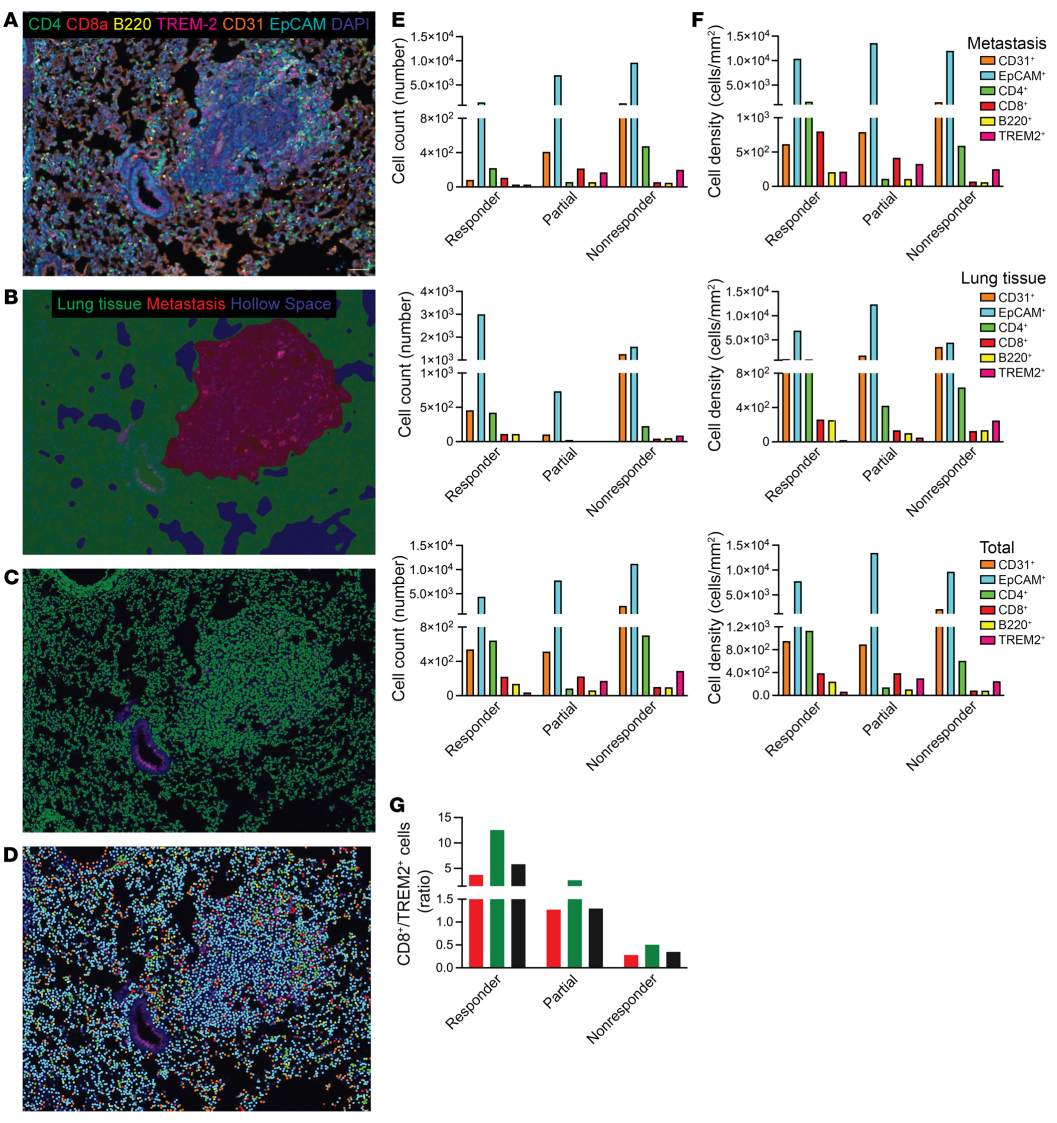
Vascular immune features reflect differential response to antiangiogenic IT. (**A**) Customized multiplexed IHC (mIHC) staining of 4T1 metastases to the lungs. An antibody panel was developed to detect CD31^+^ endothelial cells (orange), EpCAM^+^ epithelial cells (both lung epithelium and breast cancer cells, cyan), TILs (CD4^+^ T helper, green; CD8a^+^ CTLs, red; B220^+^ B-cells, yellow), and TREM2^+^ recruited TAMs (magenta). Scale bars: 50 μm. (**B**–**D**) A machine learning-based algorithm was trained to discriminate metastatic tissue (red) from lung and hollow space (green and blue, respectively; **B**), followed by cell segmentation (**C**). Phenotyping (**D**) is visualized as a dot with the same color coding as in **B**. Cells (based on DAPI detection) negative for any of the markers included in the antibody panel are displayed in blue. (**E**–**G**) total cell counts per phenotype (**E**), and cell density per phenotype (cells/mm^2^; **F**) within the different tissue segments. The CD8^+^ T cells/TREM2^+^ TAMs ratio (**G**) was calculated from cell densities.

To combine the data coming from the necropsy observations and the immunophenotyping, we developed a customized mIHC panel to delineate the spatial relationships of endothelial cells and a series of immune cell types (Figure 6A and Supplemental Table 8). In the absence of reliable reagents for the specific detection of ALK1, and to clearly distinguish the presence of different cell types, we selected CD31 to mark endothelial cells and TREM2 as a proxy to identify protumorigenic ALK1^+^ TAMs. In addition, we stained for tumor infiltrating lymphocytes (TILs): CD4^+^ T helper cells, CD8a^+^ CTLs, and B220^+^ B cells. Finally, (tumor) epithelial cells were distinguished via EpCAM. Following segmentation to classify metastatic tissue and normal/adjacent lung (Figure 6B), cell identification and phenotyping (Figure 6, C and D) mirrored the features captured with FACS, IHC, and RNA-Seq. Given the high level of heterogeneity within and between lesions, we focused on the cohort exposed to combined ALK1-Fc and immune checkpoint blockade, in which we observed a phenotypic range reflecting the overall response surveyed at sacrifice (Figure 6E), as well as for human patients undergoing IT. Metastatic tissue from a mouse that responded to therapy (based on the lung weight and metastatic count at sacrifice, as well as the H&E histology) encompassed the lowest abundance of CD31^+^ endothelial cells and TREM2^+^ TAMs (Figure 6E; responder). In contrast, a stable vasculature paralleled by an increased TAM infiltration characterized the lesion of a mouse that had a controlled metastatic burden but relapsed at the primary site (Figure 6E; partial). Finally, prominent metastatic lesion vascularization and a much higher proportion of CD4^+^ cells within TILs epitomized the group of mice that did not respond/progressed upon combined therapy administration (Figure 6E; nonresponder). This distribution was even more evident when considering the spatial density (Figure 6F): in this analysis, the responder lesion in the combination treatment group displayed the highest density of both T effector and T helper lymphocytes, together with the lowest density of endothelial cells and TAMs. The segmentation further captured that the influx of effector T cells was specific for the metastatic tissue, as the density of the CD8^+^ CTLs was comparable in the lung segments across the samples. In keeping with this, the CTL-to-macrophage ratio promptly correlated with response (Figure 6G).

Taken together, spatial analysis of the cellular composition of responding or resistant lung metastatic lesions revealed that the combination of ALK1-Fc with IT promoted an active antitumor environment, while preserving sensitivity to the antiangiogenic therapy.

### ALK1 affects the HPC niche in the BM.

**Figure 7:**
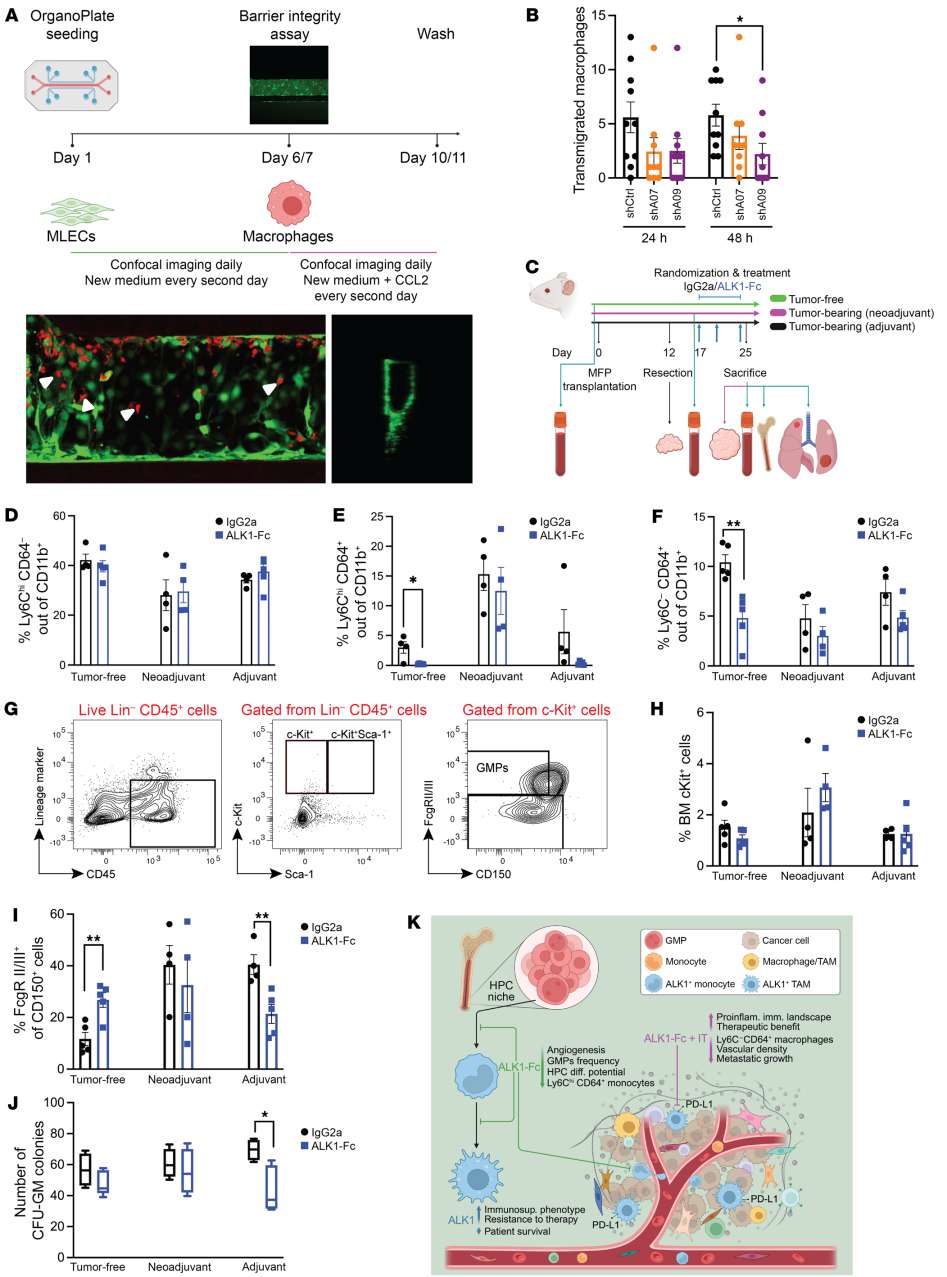
ALK1 affects the HPC niche in the BM. (**A**) Experimental design based on the organ-on-a-chip assay with 3 channels. Representative images of a longitudinal and a cross section of the 3D tube are presented in the bottom panel. (**B**) Quantification of macrophage transendothelial migration at 24 and 48 hours with endothelial cells infected with lentiviral vectors expressing either scrambled or *Acvrl1*-targeting shRNA (*n* = 4 experiments). Data are represented as mean with SEM. *P < 0.05, unpaired, 2-tailed Student’s *t* test for the comparison between shCtrl and shA07 or shA09. (**C**–**E**) Experimental design of the short-term trial based on the orthotopic transplantation of 5 × 10^4^ 4T1 cells in syngeneic BALB/c hosts (tumor-free: *n* = 5 each for IgG2a and ALK1-Fc; neoadjuvant: *n* = 4 each for IgG2a and ALK1-Fc; adjuvant: *n* = 4 for IgG2a, *n* = 5 for ALK1-Fc) (**C**). Frequency of circulating Ly6C^hi^CD64^–^ (**D**) and CD64^+^ (**E**) monocytes in peripheral blood. Data are represented as mean with SEM. (**F**) Frequency of Ly6C^–^CD64^+^ macrophages in lungs. Data are represented as mean with SEM. **P < 0.01, unpaired, 2-tailed Student’s *t* test. (**G**–**I**) FACS plot and gating strategy of c-Kit^+^Lin^–^Sca1^–^ progenitor cells (47) extracted from the BM (**G**). Frequency of c-Kit^+^ (**H**) and GMP (**I**) cells from the BM extracts. Data are represented as mean with SEM. **P < 0.01, unpaired, 2-tailed Student’s *t* test. (**J**) Quantification of colony formation plating efficiency of c-Kit–enriched BM cells. Data are represented as mean with SEM. *P < 0.05, unpaired, 2-tailed Student’s *t* test. (**K**) Drawing summarizing the findings.

Remodeling of the metastatic TME based on the response to ALK1-Fc and IT also corroborated the known and expected ALK1-dependent effects on the vasculature. Thus, we next sought to clarify whether endothelial ALK1 modulation limited the transendothelial migration of myeloid cells, as an explanation for the observed paucity of immunosuppressive TAMs in the metastatic milieu following ALK1 inhibition. To this end, murine lung endothelial cells (mLECs) engineered to express 2 different shRNA constructs against *Acvrl1* (shA07 and shA09; Supplemental Figure 6A) or a scrambled control construct (shCtrl) were seeded to populate the top channel of a 3-lane microfluidic organ-on-a-chip device (Figure 7A). A barrier integrity test confirmed the generation of an intact 3D vascular tube, allowing its perfusion with macrophages (Supplemental Figure 6B and Supplemental Video 1). In turn, the CCL2 chemoattractant in the bottom lane stimulated the macrophages to migrate to the intermediate channel. Laser scanning confocal microscopy revealed a consistent trend toward a curtailed migration through *Acvrl1*-silenced endothelium across our longitudinal imaging schedule (Figure 7B), with a significant difference for the shA09 at 48 hours, indicating that ALK1 indeed regulates extravasation of monocytes.

Subsequently, given the modulation of circulating monocyte populations observed following adjuvant ALK1 inhibition (Figure 5, N and O), we investigated whether ALK1 signaling acted upstream of macrophage functional states. We set up an in vivo experiment based on the 4T1 resection model to monitor the circulating monocytes in the framework of a short-term pharmacological inhibition of ALK1 (Figure 7C). Based on Ly6C and CD64 gating in flow cytometry analysis, we determined that ALK1-Fc reduced the frequency of circulating Ly6C^hi^CD64^+^ monocytes in tumor-free mice, and produced a similar trend in the adjuvant setting (Figure 7, D and E, and Supplemental Figure 6C). Similarly, infiltration of CD64^+^ macrophages was significantly blunted in tumor-free lungs of ALK1-Fc–treated mice compared with the IgG2a group (Figure 7F), indicating that mobilization of monocytes is under the control of ALK1 activity and independent of altered angiogenic stimuli emanating from the tumor.

Taking these results into account, we next sought to clarify which step of the hematopoietic cascade is reliant on ALK1. BM cell evaluation (47) from the experimental groups showed a significant reduction in the population corresponding to the granulocyte-macrophage progenitors (GMP) in the adjuvant ALK1-Fc group, while there was no significant change in the proportion of c-Kit^+^ progenitor cells (Figure 7, G–I). In agreement with the reduction of BM-GMPs, we also observed a significant decrease in the potential of BM-derived progenitors to differentiate ex vivo into colony-forming unit–granulocyte-macrophage (CFU-GM) colonies following exposure to adjuvant ALK1-Fc treatment in vivo (Figure 7J).

In conclusion, our data demonstrate that ALK1 functionally regulates the microenvironment in 2 specific compartments: locally, by pruning angiogenesis and fine-tuning permissiveness to monocyte/macrophage transendothelial migration; and systemically, by determining the breadth of monocyte differentiation and mobilization in the BM.

## Discussion

The data presented in our work delineate a role in macrophage biology for the hitherto endothelium-restricted ALK1 receptor (Figure 7K). Through a series of in vivo models, clinically relevant treatment schedules, mechanistic studies, and integration of in silico datasets, we uncover and validate the retained expression of ALK1 in a population of CD14^+^ monocytes in physiological and pathological breast. When zooming in on cancer, these monocytes give rise to immunosuppressive TAMs that support disease progression upon failure of therapeutic regimens. We provide compelling evidence that the monocyte-to-TAM differentiation is the final step in a cascade of events that spring from the HPC niche in the BM, where ALK1 defines the potential of early progenitor cells.

Pharmacological blockade of endothelial ALK1 signaling gained considerable attention in the mid-2010s following a series of encouraging preclinical and clinical trials in a range of different tumor types. Given the more restricted pattern of expression compared with the ubiquitous VEGF receptor 2 (VEGFR2), ALK1 inhibition became an enticing alternative to the promise of the “anti-angiogenic revolution” that ensued with the initial approval of bevacizumab. Despite this, the development of the ALK1 ligand trap dalantercept was discontinued due to the lack of additional benefits, raising the issue of the initial patient selection as well as the optimal therapeutic combination partner. In the absence of biomarkers for ALK1 status, the tumor types selected for escalation studies and phase 2 trials failed to offer much ground for the antitumor activity of dalantercept, even more so when combined with standard-of-care agents that were already suboptimal in terms of efficacy. As we were unable to access samples collected during such clinical trials, the initial indication of an active involvement of tumor immunity stems from the retrospective analysis of archival breast cancer tissue from MMTV-PyMT mice, in which neoadjuvant ALK1-Fc monotherapy promotes a proinflammatory environment, with a markedly increased expression of *Cd3* and *Cd4*, as well as factors like *Ifng* (encoding for interferon-γ) and *Cd40l*. Collectively, these markers indicate a broad rewiring of the immune cell composition and activation status as a consequence of therapeutic pressure. For example, the CD40/CD40L axis is related to B cell priming by CD4^+^ T cells, and several agonists for CD40 have surfaced for clinical validation, e.g., mitazalimab and selicrelumab.

However, addition of IT to ALK1 blockade within a neoadjuvant regimen did not translate to better tumor control in the transgenic setting. This outcome corroborates similarly to published observations of combined VEGFR2 inhibition and IT (48) and is most likely due to the low mutational burden commonly observed in genetically engineered mouse models. Nonetheless, in the context of the increasing use of neoadjuvant IT in TNBC patients, it is worth mentioning that we observed a trend toward fewer malignant foci to the lungs in any treatment group that included neoadjuvant ALK1-Fc in our mouse models (Supplemental Figure 1), suggesting that the molecular cues in the metastatic colonization and growth are only partially overlapping with those of the primary tumor mass.

Our finding of negative impact on survival of a high expression of the *ACVRL1* signature is in line with the previously reported prognostic value of protumorigenic *SPP1*^+^ TAMs (29, 34). *ACVRL1* expression was also linked to other immunosuppressive markers including FABP5 and TREM2. Notably, *TREM2* also denotes immunosuppressive (and M2-like) TAMs with a putative monocytic origin (49, 50). Moreover, the ability of ALK1 to directly specify an immunosuppressive phenotype of TAMs by regulating the transcription of *CD274* endows an unprecedented opportunity to investigate additional partners that could help sustain these immunomodulatory properties. Our proof-of-concept trial unequivocally indicates PD-1 and CTLA-4 inhibition as actionable therapeutic companions to ALK1 inhibition. An additional line of evidence that supports this combination comes from a clinical trial promoted by the pharmaceutical company Kintor, which resumed the development of GT-90001, a monoclonal antibody against ALK1 originally pursued by Pfizer. This early phase investigation determined the recommended dose of GT-90001 together with the anti–PD-1 nivolumab in advanced hepatocellular carcinoma (51). Except for thrombocytopenia, this combination showed a good safety profile, tolerability, and antitumor activity to justify further investigation. Our work provides a mechanistic rationale to further pursue the combination of ALK1 inhibition with immune checkpoint blockade clinically. However, it should be noted that the mode of action of ALK1-Fc is not necessarily the same as that of GT-90001; whereas ALK1-Fc will leave the native receptor available for binding of low-affinity ligands, such as TGF-β, GT-90001 will leave high-affinity ligands BMP9/10 the opportunity of binding other TGF-β type I receptors, e.g., ALK2.

The yield brought in by combined blockade of ALK1 and immune checkpoints reflects an intra-metastatic, as well as a systemic, underrepresentation of an immunosuppressive arm of the immune compartment, culminating with a depletion of recruited TAMs and circulating monocyte-derived cells, respectively. Even our bulk RNA-Seq captures this macrophage-centered effect, as ALK1-Fc downregulates interferon-induced transmembrane (IFITM) 6 transcripts. The IFITM family of proteins is involved in cell-cell adhesion and cell differentiation; IFITM6 is specifically expressed in BM-derived macrophages and its expression is increased in tumor-bearing mice (52). The finding that ALK1 inhibition only reduced metastatic burden, but did not affect metastatic incidence in the E0771 model, is most likely attributable to the fact that treatment was started when mice were already in advanced stage breast cancer, i.e., with preexisting disseminated disease. Unlike the E0771-based system, in which only 40% of the mice generated macro-metastases, the 4T1 cell line gives rise to tumors resembling stage IV human TNBC with a complete penetrance to different metastatic sites in a time-dependent manner. Despite the more aggressive growth pattern and metastatic penetrance of the 4T1 syngrafts, the addition of IT to ALK1 inhibition specifies for a higher frequency of NK cells and CD4^+^ T cells as well as dendritic cells. Globally, our in vivo approaches confirm a boosted infiltration and functionality of T helper, CTLs, B cells, and professional antigen-presenting cells following exposure to ALK1-Fc–encompassing therapies. These cell types are the major constituents of tertiary lymphoid structures (TLSs), which have been characterized as prime determinants of sustained antitumor activity in patients treated with IT (53). Although protocols for the detection and spatial characterization of TLS have been standardized and are widely available for clinical investigation, translation to murine models is not trivial, as these agglomerates cannot be generally observed in full (54). It becomes even more cumbersome in the metastatic setting, which suffers from the limited availability of data from the clinical experience (55). In agreement with this, the full-slide mIHC panel we customized for breast dissemination to the lungs did not reveal the presence of TLS in the metastases or embedded within the adjacent lung tissue, except for one single lesion displaying an interesting germinal center-like core in a lymphocyte-rich structure. Given the dual nature of ALK1 expression in both blood vessels and TAMs, it is tantalizing to speculate about an active role of this signaling pathway in dampening the antitumor potential offered by TLS. In support of this idea, administration of ALK1-Fc significantly upregulated the expression of *Tnfsf14*/LIGHT, an essential chemokine that drives the formation of high endothelial venules (HEVs) and TLS (56). This modulation underlies that HEVs may not respond to conventional proangiogenic and prolymphangiogenic signals for their genesis; thus, whether ALK1 is functionally implicated in the development of HEVs remains to be established.

A proportion of mice in our in vivo 4T1 setup did progress even in the combined treatment group, reminiscent of the well-known heterogeneity of response to IT in patients. In this respect, inspection of the longitudinal scRNA-Seq human melanoma dataset (41) confirms that *ACVRL1* expression follows that of PD-L1 and PD-L2. Interestingly, *Pdcd1lg2*/PD-L2 was consistently upregulated following ALK1 inhibition in the MMTV-PyMT model. The exact role of PD-L2 in respect to PD-L1 is still unclear, with mounting evidence about immune-independent functions of PD-L2 adding to the debate (57). Our experimental data do not seem to support a coordinate expression and utility of these 2 ligands, as for example SMAD5 is seemingly involved in the transcriptional control of *CD274* only but not *PDCD1LG2*, despite their genomic proximity. From the clinical perspective, this large melanoma cohort also validates that ALK1 expression in TAMs correlates with resistance, with important clinical implications in terms of eligibility of treatment. This observation is particularly relevant in the search for additional companion targets to ALK1 inhibition. For example, *HAVCR2*/TIM-3 is expressed by T cells to enforce a crucial immune checkpoint function associated with acquired resistance to anti–PD-1 therapy in lung adenocarcinoma (58). Ongoing clinical trials are evaluating the efficacy of agents against TIM-3: while most of the expected effects derive from lymphocytes, these studies should expand to a broader immune landscape, with a thorough characterization of both T cell–dependent and –independent functions (59). In consideration of our in vivo results describing a rewired immune landscape, including an increased frequency of T cells, further studies to assess the role of TIM-3 inhibition in the framework of ALK1 blockade are warranted.

Finally, with the aim of providing an optimal patient stratification for therapeutic gain, it will be paramount to address the concordance between endothelial and myeloid ALK1 expression.

Our transplantation-based approach limits us in uncoupling the specific contribution of ALK1 signaling in the endothelial versus myeloid compartment. To circumvent this, we devised more controlled strategies to dissect these distinct aspects of ALK1 biology. First, the exploitation of the organ-on-a-chip platform recapitulates the intertwined relationship between tissue vascularization and immune cell trafficking. As the depletion of *Acvrl1* in the endothelial compartment largely hinders the migration of macrophages, it is likely that modulation of ALK1 remodels the expression of adhesion molecules involved in the multistep migration of leukocytes. In agreement with this, expression of *Selp* (encoding for P-selecting, necessary for tethering and rolling of leukocytes) is downregulated in liver sinusoidal endothelial cells lacking ALK1 (60). Given the appreciable reduction in the proportion of CD64^+^ macrophages in the lung enhanced by combining IT with ALK1-Fc, these data suggest that ALK-mediated remodeling of the leukocyte migration pattern directly affected the circulating monocyte population. Second, to further dismiss the confounding effect of ALK1 at sites of neoangiogenesis, the administration of the ALK1 ligand trap in tumor-free hosts exposes the importance of this signaling pathway in the BM. Interestingly, our short-term trial in the 4T1 model corroborates the trends observed in trials with a longer duration but expanded on the role of ALK1 in the mobilization of monocytes, as evinced by the evidently lower relative number of macrophages in tumor-free hosts in the ALK1-Fc cohort compared with the IgG2a control cohort. This regulation transcends the specific requirements dictated by tumor evolution and holds a broader relevance for pathologies that rely on hematopoietic dysfunction for their medical evolution, including the human hemorrhagic telangiectasia that is caused by congenital mutations in *ACVRL1*. An additional readout is the different magnitude of response based on the modality of treatment: no effect was appreciable in our neoadjuvant setting, reinforcing the vital role of primary tumor-derived immunosuppressive signals and crucial differences between the primary tumor ecosystem and metastatic lesions (26). In support of this, recent clinical evidence suggests a greater IT efficacy when administered during early stages of TNBCs and before the activation of the immunosuppressive mechanism that characterizes later stages (61). Furthermore, promotion of an immunosuppressive TME is a preserved feature of growing malignant masses, as our data indicate that progenitor cells extracted from the BM of the MMTV-PyMT model are also more efficient at monocytopoiesis than progenitors from nontransgenic littermates (Supplemental Figure 6D).

In the field of TGF-β–related biology, ALK1 has only recently abandoned the role of a pure mediator of endothelial identity and function in favor of a more multifaceted player in different homeostatic processes and diseases, including normal hair maintenance, pulmonary hypertension, and atherosclerosis (62–65). An additional layer included in our study concerns the differentiation potential of HPCs and the ability of ALK1 to selectively determine the fate of GMPs during tumor evolution. The observed reduction in the GMP population upon ALK1-Fc treatment is evocative of the pattern evinced from the BM atlas (28) (Supplemental Figure 2C), which singled out the highest average expression of *ACVRL1* in the GMP cluster. In this context, it remains to be ascertained whether a blunted ALK1 signaling is a priming event that is retained in the differentiation cascade of these early progenitors or if it can be reverted. Finally, the bone represents the most common metastatic site in nonbasal breast cancer. Given the peculiar macrophage signature recently uncovered in patients diagnosed with luminal subtypes (66), future efforts will also aim to define the requirement for ALK1 signaling in bone tropism of cancer cells as well as the conceivable interplay between the hematopoietic and metastatic niches during tumor evolution. As cancer IT is rapidly incorporating myeloid modulation, a new wave of clinical trials has projected their efforts in reprogramming and reeducating macrophage activity through ex vivo polarization, adoptive transfer, engineering, and chimeric antigen receptor–macrophage (CAR-macrophage) approaches (67, 68). In contrast, our work underscores the potential of ALK1 as a target to act upstream of protumorigenic myeloid infiltration in the tumor mass and thereby ameliorate therapeutic response.

In conclusion, our data shed light on previously unknown features of ALK1 signaling underpinning the clinical value of ALK1 suppression to promote a multifaceted antitumor activity. In anticipation of a revamped interest in resuming the development of pharmacological inhibitors against this receptor, our data warrant additional investigation to deconstruct the stromal hierarchy embedded in ALK1 signaling for therapeutic gain.

## Methods

### Sex as a biological variable.

Our study exclusively examined female mice because the disease modeled is mostly relevant in females, as no less than 99% of breast cancer diagnoses occur in women versus men.

For further information, see Supplemental Methods.

### Data availability.

The RNA-Seq data have been deposited in the NCBI’s Gene Expression Omnibus database (GEO GSE26091). Values for all data points in graphs are reported in the Supporting Data Values file.

### Statistics.

Statistical analyses were performed as indicated in the figure legends and in the Methods section of the supplementary material.

### Study approval.

All mouse work was performed according to the permits M167-15 and 14122-2020 approved by the local ethical committee for animal experimentation.

## Author contributions

MST, JS, PB, EK, TVP, S Larsson, S Lehn, GF, VI, SB, and MB developed new methodology. JS, PB, and ML wrote new software. MST, JS, PB, S Lehn, MB, and KP performed validation of data. MST, JS, PB, EK, TVP, S Larsson, CO, ML, GJ, CR, MB, and KP performed formal analysis. MST, JS, PB, EK, EC, TVP, S Larsson, GF, VI, SB, JP, and MB performed experimental investigations. RSP and KP provided resources. MST, JS, PB, EK, MB, and KP curated data. MB and KP wrote the original draft of the manuscript. MST, JS, PB, EK, EC, TVP, S Larsson, S Lehn, GF, VI, SB, JP, CO, ML, RSP, GJ, and CR reviewed and edited the manuscript. MST, JS, PB, MB, and KP provided visualization of the data. MB and KP supervised the project. MB and KP administered the project.

## Supplementary Material

Supplemental data

Supplemental table 1

Supplemental table 2

Supplemental table 3

Supplemental table 4

Supplemental table 5

Supplemental table 6

Supplemental table 7

Supplemental table 8

Supplemental video 1

Supporting data values

## Figures and Tables

**Table 2:**
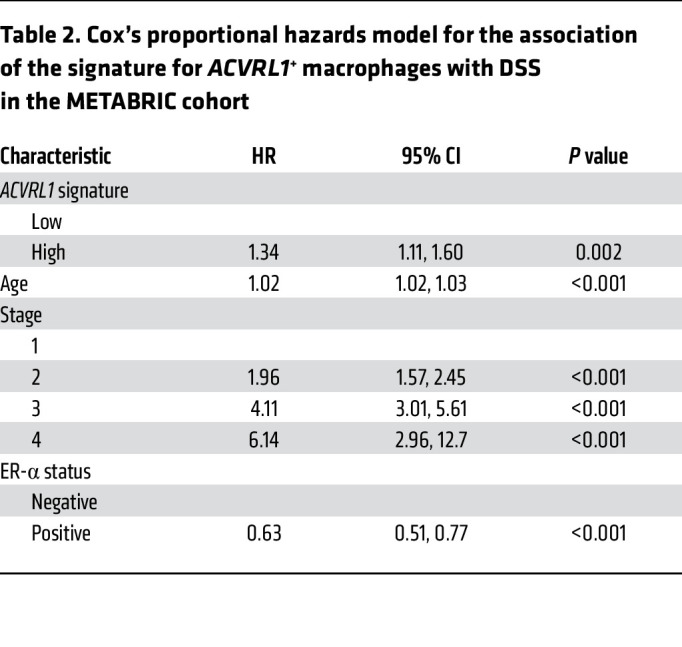
Cox’s proportional hazards model for the association of the signature for *ACVRL1*^+^ macrophages with DSS in the METABRIC cohort

**Table 1:**
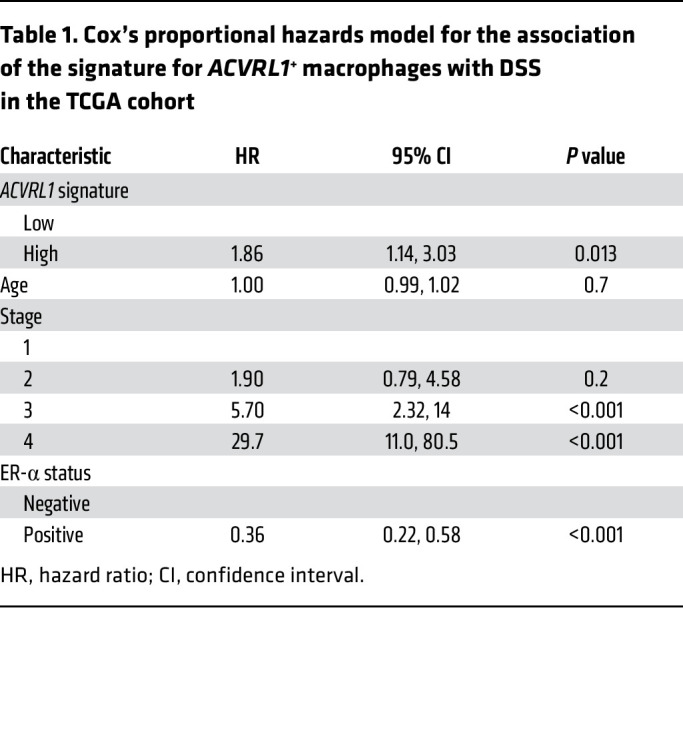
Cox’s proportional hazards model for the association of the signature for *ACVRL1*^+^ macrophages with DSS in the TCGA cohort

## Version 1 01/14/2025

In-Press Preview

## Version 2 03/03/2025

Electronic publication
